# HbA1c variability and long-term glycemic control are linked to peripheral neuropathy in patients with type 1 diabetes

**DOI:** 10.1186/s13098-020-00594-4

**Published:** 2020-10-06

**Authors:** M. V. Pinto, L. C. G. F. Rosa, L. F. Pinto, J. R. Dantas, G. F. Salles, L. Zajdenverg, M. Rodacki, M. A. Lima

**Affiliations:** 1 Neurology Section, Federal University of Rio de Janeiro, Rio de Janeiro, Brazil; 2 Department of Neurology, Mayo Clinic, Rochester, MN USA; 3 Nutrology and Diabetes Section, Federal University of Rio de Janeiro, Rio de Janeiro, Brazil; 4 Department of Internal Medicine, Federal University of Rio de Janeiro, Rio de Janeiro, Brazil

**Keywords:** Diabetic peripheral neuropathy, Type 1 diabetes, Long-term glycemic control

## Abstract

**Background:**

HbA1c variability has been linked to retinopathy, renal disease and autonomic neuropathy in patients with type 1 diabetes mellitus (T1D) and type 2 diabetes mellitus (T2D). Although the same relationship has been demonstrated for diabetic peripheral neuropathy (DPN) in patients with T2D, data for T1D are still lacking.

**Methods:**

Patients older than 17 years of age with ≥ 10 years of T1D duration and follow-up were included. All patients underwent nerve conduction studies and neurological examination. Laboratorial data was retrospectively extracted from chart review. Mean HbA1c (mHbA1c) over 10 years was calculated, as well as HbA1c variability estimated by standard deviation (HbA1c-SD) and coefficient of variation (HbA1c-CV).

**Results:**

Fifty patients with T1D were included (30 females and 21 non-caucasians), with mean age and T1D duration of 25.6 ± 5.0 and 17.9 ± 6.1 years, respectively. The frequency of DPN was 24%. Higher mHbA1c (10.4 ± % vs 8.1 ± %; p < 0.001), HbA1c-SD (1.8 ± 0.8 vs 0.9 ± 0.4; p < 0.001), and HbA1c-CV (1.7 ± 0.8 vs 1.2 ± 1.1; p = 0.006) were observed in patients with DPN compared to others. SD-HbA1c and HbA1c-CV were associated with DPN, diagnosed by either clinical or NCS criteria, independent of mHbA1c, age and gender.

**Conclusions:**

Not only long-term glycemic control, but also its variability is associated with DPN in patients with T1D. Larger studies are required to confirm this finding.

## Introduction

Long-term glycemic control is a well-known risk factor for diabetic chronic complications [1], but there is recent evidence that glucose variability (GV) may also play a role in their development [2].

GV can be interpreted as intraday or interday glucose variation or long-term variation of HbA1c, which can be assessed by standard deviation (HbA1c-SD) and coefficient of variation (HbA1c-CV) [3]. Short-term GV measured through indices such as mean amplitude glucose excursions (MAGE) has been associated with microvascular complications in patients with type 2 diabetes (T2D), but their relationship is less clear for type 1 diabetes (T1D) [4, 5]. HbA1c variability has been linked to retinopathy, renal disease and autonomic neuropathy in patients with T1D and T2D [6–8]. Although the same relationship has been demonstrated for diabetic peripheral neuropathy (DPN) in patients with T2D [9], data for T1D are still lacking. Our aim was to investigate the association between DPN and HbA1c-SD or HbA1c-CV in individuals with T1D.

## Methods

In this cross-sectional study, patients from the Federal University of Rio de Janeiro’s Diabetes clinic older than 17 years of age with ≥ 10 years of T1D duration and follow-up were included. Ethics Committee approved the study and subjects signed an informed consent. Laboratorial data was obtained through review of medical charts. HbA1c was evaluated with NGSP certified method HPLC with coefficient of variation < 2% (Biorad USA). The results of all HbA1c measurements available were used to calculate the mean HbA1c (mHbA1c) and HbA1c variability over 10 years. HbA1c variability was estimated by HbA1c-SD (standard deviation of the mHbA1c) and HbA1c-CV (HbA1c-CV = HbA1c − SD/[0.1 × mHbA1c]).

Between 2014 and 2015, patients underwent neurological examination performed by a neurologist (L.F.P.) and nerve conduction studies (NCS) performed by an experienced neurophysiologist (M.V.P.). DPN was diagnosed by symptoms or signs associated with abnormalities on NCS in sural and an additional nerve (Toronto Diabetic Neuropathy Expert Group criteria) [10]. Motor NCS were performed on median, ulnar, peroneal and tibial nerves with determination of conduction velocity, amplitude, distal motor latency, and F-waves (except for peroneal). Sensory NCS were performed on median, ulnar, superficial peroneal and sural following standard techniques [11]. NCS were done on non-dominant upper extremity and right leg.

Neuropathy symptoms score (NSS) and neuropathy disability score (NDS) evaluations were performed in all patients. Explanation of both scores is provided elsewhere [12, 13]. Briefly, NSS is a score of neuropathic symptoms that varies from 0–9, and is subdivided as mild (score 3–4), moderate (5–6) and severe (7–9) symptoms. NDS is a score of neuropathic findings on exam that varies from 0–10, and is subdivided as mild (score 3–5), moderate (6–8) and severe (9–10) signs of neuropathy. The minimum acceptable criteria for a diagnosis of peripheral neuropathy are: moderate signs with or without symptoms, or mild signs with moderate symptoms.

We compared NSS/NDS criteria (NSS/NDS-PN) to symptoms and/or signs plus abnormal NCS. Sensitivity, specificity, positive predictive value (PPV) and negative predictive value (NPV) of NSS/NDS-PN for diagnosis of DPN were calculated.

Statistical analysis was performed using IBM SPSS 23.0 STATISTICS. For continuous measurements, results were expressed as mean and SD. Chi-square and Fisher tests were used to test associations between qualitative variables. Mann Whitney was used for continuous and nominal variables. Multivariate logistic regression was used to assess independent associations between mHbA1c, HbA1c-SD or HbA1c-CV with DPN ascertained by clinical criteria (NSS/NDS), NCS and or both. Multivariate analyses were first adjusted for age and sex (Model 1) and then further adjusted for BMI, and for the presence of essential hypertension and of other microvascular complications (Model 2). The results were presented as odds ratios (ORs) with their 95% confidence intervals (CIs). To allow comparisons between mHbA1c and HbA1c variability in their strengths of association with DPN, ORs were standardized for increments of 1-SD in each HbA1c parameter. A 2-tailed probability value < 0.05 was considered significant.

## Results

Fifty patients with T1D were included (30 females and 21 non-caucasians), with mean age and T1D duration of 25.6 ± 5.0 and 17.9 ± 6.1 years, respectively. The mean number of HbA1c measurements/year and mean-HbA1c throughout follow-up were 2.5 ± 1.1 and 8.7 ± 1.7%. The majority (56%) of patients were using human insulin (NPH) as basal insulin while 40% used long-acting insulin analogs and 4% used continuous system of insulin infusion. For bolus injections, short-acting insulin analogues were used in 50% of the cases and regular insulin in the other half of patients. The frequency of DPN was 24% (12/50 patients). Sixteen patients fulfilled combined NSS/NDS criteria for peripheral neuropathy (NSS/NDS-PN), of which 11 were from the DPN group (Toronto criteria) and 5 from the non-DPN group. Mean NSS and NDS scores were higher in DPN than in non-DPN group (4.2 ± 3.2 vs 0.6 ± 1.8; p < 0.001 and 7.2 ± 2.0 vs 1.4 ± 2.1; p < 0.001 respectively). Sensitivity, specificity, PPV and NPV of NSS/NDS-PN for DPN were 91.7%, 86.8%, 68.8% and NPV 97.4%, respectively.

Higher mHbA1c (10.4 ± % vs 8.1 ± %; p < 0.001), HbA1c-SD (1.8 ± 0.8 vs 0.9 ± 0.4; p < 0.001), and HbA1c-CV (1.7 ± 0.8 vs 1.2 ± 1.1; p = 0.006) were observed in patients with DPN compared to others. SD-HbA1c and CV-HbA1c were associated with DPN, diagnosed by either clinical or NCS criteria, independent of mHbA1c and other potential confounders (Table 1). Moreover, strengths of associations were higher for HbA1c variability than for mHbA1c, except for DPN diagnosed by NCS.

**Table 1:** Results of multivariate logistic regression for the associations between HbA1c parameters (mean and variability) and presence of peripheral neuropathy by clinical and nerve conduction studies criteria

HbA1c parameters	Diabetic peripheral neuropathy diagnosis					
	Clinical (NSS/NDS) n = 16		Nerve conduction studies n = 14		Clinical and NCS (Toronto criteria) n = 12	
	Model 1* OR (95% CI)	Model 2* OR (95% CI)	Model 1* OR (95% CI)	Model 2* OR (95% CI)	Model 1* OR (95% CI)	Model 2* OR (95% CI)
HbA1c-SD	3.65 (1.11–12.05)^†^	3.77 (0.98–14.49)	4.22 (1.04–17.24)^†^	4.88 (0.83–28.57)	10.41 (1.38–78.4)^†^	13.44 (1.46–123.6)^†^
Mean HbA1c	2.21 (0.84–5.81)	2.07 (0.72–5.95)	12.35 (1.74–83.3)^†^	10.10 (1.14–90.9)^†^	4.99 (0.92–27.16)	3.91 (0.83–18.34)
HbA1c-CV	2.75 (1.06–7.09)^†^	2.75 (0.95–8.00)	3.38 (1.04–10.99)^†^	3.83 (0.83–17.86)	6.57 (1.25–34.72)^†^	8.47 (1.25–57.61)^†^
Mean HbA1c	3.26 (1.28–8.26)^†^	3.08 (1.12–8.47)^†^	20.0 (2.77–142.8)^‡^	16.67 (2.09–142.9)^‡^	10.79 (1.93–60.2)^‡^	8.59 (1.62–45.61)^†^

*NSS* neuropathy symptoms score, *NDS* neuropathy disability score, *NCS* nerve conduction study, *OR* odds ratio, *CI* confidence interval

* Odds ratios were estimated for increments of 1 − SD in each HbA1c parameter (mean HbA1c 1.7%, HbA1c-SD 0.7% and HbA1c-CV 6.3), and adjusted for age and sex in Model 1 and further adjusted for BMI, presence of essential hypertension and of other microvascular complications in Model 2. ^†^p < 0.05; ^‡^p < 0.01

## Discussion

This study demonstrates the association between DPN and long-term HbA1c variability in patients with T1D. GV can activate overproduction of oxygen reactive species, increase production of inflammatory cytokines, induce cell apoptosis and stimulate epigenetic changes that might increase the risk of DPN [14]. Therefore, a modern approach for glycemic control should focus not only on HbA1c levels, but also on GV [15].

Virk et al. were the first to study the influence of HbA1c variability for development of DPN and cardiac autonomic neuropathy (CAN), in a group of young patients with long-standing T1D [16]. HbA1c variability was associated with CAN but not with DPN. Moreover, the classic association of DPN with CAN was not present. These results were unexpected but DPN was diagnosed only with quantitative sensory testing (QST) abnormalities in thermal or vibratory threshold [16]. QST was not included in either the definition criteria of distal symmetric polyneuropathy of American Academy of Neurology [17] or Toronto expert panel in DPN due to high variability in sensibility and specificity and difficult standardization and reproducibility [10]. Therefore, those results may be related to the selection of DPN criteria. We used symptoms, signs and NCS abnormalities, making the diagnosis of DPN more accurate and robust. Recently, Su et al., with similar diagnostic criteria, showed that increased HbA1c variability is closely associated with DPN in patients with T2D [18].

Our study has limitations. We did not exclude patients with less than two HbA1c measurements per year. However, the mean number of HbA1c measurements was comparable to other longitudinal studies [19, 20]. We did not use a specific method to evaluate small sensory nerve fibers rather than neurological examination. In addition, short-term GV was not evaluated. Finally, the small sample size prevented more comprehensive statistical adjustments. Nonetheless, this was the first study to demonstrate the association of HbA1C variability over time and DPN in T1D patients.

## Conclusion

Not only long-term glycemic control, but also its variability is associated with DPN in patients with T1D. Larger studies are required to confirm this finding.

## Data Availability

The datasets used and/or analysed during the current study are available from the corresponding author on reasonable request.

## Abbreviations

DPN: Diabetic peripheral neuropathy
GV: Glucose variability
mHbA1c: Mean HbA1c in the study period (2006–2015)
NSS: Neuropathy symptom score
NDS: Neuropathy disability score
NSS/NDS-PN: Combined NSS/NDS criteria for peripheral neuropathy
HbA1c-SD: Standard deviation of all HbA1c measurements during the study period (2006–2015)
HbA1c-VC: Variation coefficient of all HbA1c measurements during the study period (2006–2015)
T1D: Type 1 diabetes mellitus
T2D: Type 2 diabetes mellitus
